# Plasmin–Plasminogen System and Milk Physicochemical Traits in Intensively Reared Chios and Frizarta Ewes: Effects of Lactation Stage, Age, and Somatic Cell Count

**DOI:** 10.3390/ani16071041

**Published:** 2026-03-28

**Authors:** Aphrodite I. Kalogianni, Eleni Dalaka, Georgios Theodorou, Ioannis Politis, Athanasios I. Gelasakis

**Affiliations:** Department of Animal Science, School of Animal Biosciences, Agricultural University of Athens (AUA), 11855 Athens, Greece; afrokalo@aua.gr (A.I.K.); elenidalaka@aua.gr (E.D.); gtheod@aua.gr (G.T.); i.politis@aua.gr (I.P.)

**Keywords:** plasmin, plasminogen, somatic cell count, electrical conductivity, refractive index, pH, body condition score, dairy sheep, Chios breed, Frizarta breed

## Abstract

The objective of the present study is to evaluate the effects of lactation stage, age, somatic cell count (SCC), and daily milk yield on plasmin–plasminogen (PL–PG) system activity and physicochemical milk traits in intensively reared Chios and Frizarta ewes. A total of 52 purebred ewes (26 ewes per breed and farm) were randomly selected and prospectively monitored during the 3rd, 5th, and 6th month post-lambing. Daily milk yield and body condition score (BCS) were recorded, and individual milk samples were collected for the assessment of PL–PG activities using enzymatic assays, SCC, electrical conductivity (EC), refractive index (RI), and pH. Correlation analysis and mixed linear regression models were used for the assessment of the effects. Lactation stage significantly affected PL–PG system traits in both breeds, but in opposite direction; plasmin and plasminogen plus plasmin declined toward late lactation in Chios ewes, whereas it increased in Frizarta ewes. Lower SCC was associated with reduced plasmin system activity in Chios ewes, whereas no effect was observed in Frizarta ewes. The plasminogen-to-plasmin ratio remained stable across lactation, breeds, and SCC classes, indicating coordinated regulation of the system. BCS was positively associated with plasmin activity during late lactation, suggesting a stage-dependent metabolic modulation. EC and pH were closely associated with SCC, while RI mainly reflected compositional variation. Our findings underline that, although the PL–PG system is primarily affected by lactation stage and mammary health status in sheep, there are breed-specific regulatory patterns which should be further investigated.

## Simple Summary

The plasmin–plasminogen (PL–PG) system is the main endogenous proteolytic pathway in milk and plays an important role in mammary gland physiology, milk quality, and cheese-making properties. In dairy sheep, information regarding the regulation of this system across lactation and between breeds remains limited. This study aims to assess the effects of lactation stage, age, and somatic cell count on the PL–PG system, electrical conductivity, refractive index, and pH in Frizarta and Chios ewes during the mid- to late-lactation period. Lactation stage was significantly associated with the PL-PG system with different directions in two breeds. Also, body condition score and somatic cell counts were positively associated with plasmin activity, especially in Chios ewes. Daily milk yield had no consistent effect on plasmin system activity. Electrical conductivity and pH were positively associated with SCC, while the refractive index did not present a consistent response to any of the factors studied. The results highlight that the PL-PG system is primarily affected by lactation stage and mammary health status, while breed-specific physiological differences may also influence endogenous proteolysis patterns in ovine milk.

## 1. Introduction

Extracellular proteolysis during tissue remodeling is mediated by tightly regulated proteolytic systems, among which the plasmin–plasminogen (PL–PG) system plays a central role. This system involves the conversion of the inactive glycoprotein plasminogen to the active serine protease plasmin under the action of plasminogen activators (tissue type and urokinase type), which are produced by various cell types under tight regulatory control and in response to diverse extracellular signals [1,2]. In milk, plasmin constitutes the major endogenous protease and is primarily responsible for the hydrolysis of αs- and β-caseins, thereby directly affecting milk protein stability, technological properties, and cheese yield [3,4].

In dairy cows, activation of the PL–PG system has been widely recognized as a hallmark of post-lactation mammary involution. Plasmin activity increases progressively toward late lactation and the dry period, reflecting enhanced epithelial permeability, increased transfer of blood-derived plasminogen, and upregulation of plasminogen activator activity [5]. Beyond its physiological role in involution, activation of the PL–PG system in bovine milk has also been associated with inflammatory processes affecting mammary gland integrity, particularly mastitis, where excessive proteolysis contributes to the deterioration of milk coagulation properties and reduced cheese-making performance [4,6].

Contrary to dairy cows, information on the regulation and biological significance of the PL–PG system in dairy sheep remains comparatively limited. Available studies indicate that plasmin activity in ewe milk is influenced by the somatic cell count (SCC) and stage of lactation; however, reported patterns are often inconsistent across breeds and production systems. Albenzio et al. [7,8] demonstrated that an increased SCC and advancing lactation are associated with elevated plasmin activity and impaired cheese-making properties in ewe milk, whereas other studies have reported breed-dependent or flock-specific responses. Also, Koutsouli et al. [9] report that PL-PG activity declines during late lactation and increases in association with elevated SCC. Rebucci et al. [10] further highlight a strong association between SCC and plasminogen activator activity in sheep and goat milk, supporting the role of endogenous proteolysis as an indicator of mammary gland health in small ruminants.

Breed effects have emerged as an additional source of variability in the regulation of the PL–PG system in sheep. Theodorou et al. [11], in a comparative study of Greek dairy sheep breeds with major differences in milk production capacity, reported significant effects of breed, lactation stage, and udder health status on plasmin and plasminogen-derived activity. Importantly, that study suggests that part of the between-breed variation in PL–PG system traits could be attributed to differences in genetic potential for milk yield. Nevertheless, it remains unclear whether breed-dependent differences in PL–PG system regulation persist among highly productive sheep breeds reared under comparable intensive management conditions, independently of production level per se.

Assessment of the PL–PG system in milk cannot be fully interpreted without a concurrent evaluation of mammary gland health indicators and physicochemical milk traits. Somatic cell count is widely used as an indicator of mammary inflammation and increases as a consequence of innate immune activation and epithelial cell damage. Electrical conductivity (EC) of milk is an intrinsic physicochemical trait closely associated with mastitis, increasing as a consequence of disrupted tight junction integrity and altered ion transport, leading to elevated sodium and chloride concentrations in milk [12]. Milk pH is also known to increase during inflammatory conditions; although its diagnostic performance for subclinical mastitis is limited, particularly in dairy cows [13], pH remains a relevant factor affecting enzyme activity, casein micelle stability, and coagulation properties [14]. The refractive index (RI) has also been proposed as a reliable predictor of milk fat, protein, lactose, and total solids content in ovine milk, especially when combined with EC and pH measurements, providing a practical tool for on-farm milk quality assessment [15]. Changes in the refractive index may therefore reflect compositional shifts occurring during lactation or under inflammatory conditions, which can indirectly influence the physicochemical environment in which endogenous proteolytic systems operate.

In dairy sheep, several studies have documented the influence of lactation stage and SCC on milk composition, physicochemical properties, and cheese-making properties. Pavić et al. [16] and Bianchi et al. [17] reported marked changes in milk pH, ionic balance, and composition throughout lactation, while Jaeggi et al. [18], Revilla et al. [19], and Paschino et al. [20] demonstrate that elevated SCC adversely affects milk protein profile, coagulation properties, and cheese-making performance across different sheep breeds. Beyond the plasmin system, additional indigenous proteolytic enzymes contribute to milk protein turnover in ovine and caprine milk, interacting with inflammatory and physiological signals during lactation [21]. These findings underline the need for an integrated approach when investigating endogenous proteolysis in ovine milk.

In Greece, Chios and Frizarta sheep represent two of the most productive indigenous dairy sheep breeds and are among the most widely preferred by local farmers. The Chios breed was historically reared under small-scale traditional production systems, which facilitated its early dissemination and gradual incorporation into organized breeding and genetic improvement programs. Today, Chios sheep are predominantly managed under intensive dairy production systems, reflecting their high milk yield potential and adaptability to modern management practices. In contrast, the Frizarta breed was developed more recently through systematic crossbreeding and selection and has been primarily associated with intensive dairy production systems since its establishment. Owing to their high-yielding capacity, both breeds have been extensively used for genetic improvement purposes, and purebred or crossbred breeding stocks are commonly reared under intensive, zero-grazing management schemes. Milk from these breeds is primarily destined for the manufacture of Protected Designation of Origin cheeses, particularly Feta, underlining their strategic importance for the Greek dairy sheep sector [22].

Although milk production traits of Chios and Frizarta sheep have been studied and, in some cases, systematically recorded within breeding and selection programs, research has mainly focused on general physicochemical milk characteristics. In contrast, traits directly related to endogenous proteolysis, cheese-yield potential, and mammary gland functional status have received comparatively limited attention. Consequently, information describing PL–PG system activity patterns in these two breeds remains scarce. While the regulation of the PL–PG system in the milk of the Chios breed has been addressed in previous studies [11], corresponding data for Frizarta sheep are, to our knowledge, lacking. This knowledge gap, together with inconsistent findings reported across different sheep breeds and production systems, restricts the extrapolation of generalized conclusions and highlights the need for evidence regarding the existence of breed-specific PL–PG system activation patterns.

To address these research gaps, two hypotheses were tested. The first hypothesis is that lactation stage, age (lactation number), and SCC are associated with the PL–PG system as well as with EC, RI, and pH of ovine milk. The second hypothesis is that breed-specific differences exist under intensive dairy sheep management conditions with respect to these traits during lactation. Accordingly, the objective of the present study is to prospectively quantify plasmin and plasminogen activity and selected physicochemical milk traits in Chios and Frizarta ewes, to compare breed-specific patterns, and to evaluate their associations with lactation stage, age, and SCC, providing, to our knowledge, the first characterization of PL–PG system activity in the milk of Frizarta sheep.

## 2. Materials and Methods

### 2.1. Farms and Animals of the Study

Two intensive commercial dairy sheep farms rearing purebred Frizarta (Farm A) and Chios (Farm B) sheep were involved in the study. The two farms are located at the region of Aetolia-Acarnania (Western Greece) and were selected based on their similar structure and operation, as well as their typical intensive management scheme (feeding, reproductive and health management were scheduled in both farms by the same consultants). The characteristics of the two farms are summarized in Table 1. All ewes from the two farms at postweaning were milk-measured and clinically examined and a total of 52 healthy purebred multiparous (2nd, 3rd, and 4th parity) milking ewes (26 Frizarta and 26 Chios) with similar mean daily milk yield (1.35 L for Frizarta and 1.45 L for Chios ewes) were selected and enrolled in a 4-month prospective study. Sample collection and data recording were performed at three time points for each animal, namely during the 3rd, 5th, and 6th month post-lambing, representing the mid- to late-lactation period.

### 2.2. Milk Sampling and Analyses

At each sampling occasion, quantitative and qualitative milk analyses were applied for each ewe, including milk yield recording and the estimation of plasmin and plasminogen activities, EC, RI, pH, and SCC. During the milk yield recording procedure and for the collection of representative milk samples for the laboratory analyses, an ICAR (International Committee of Animal Recording)-approved milkmeter was used (Waikato Milkmeter, InterAg, Hamilton, New Zealand). Two 70 mL samples per ewe were collected directly from the milkmeter. Sodium azide (Sodium azide tablets, Supelco^®^, Merck Millipore, Darmstadt, Germany) was added only to the first set of samples prior to transfer, and both sets of samples were stored at 4 °C and delivered to the laboratory for analyses within 24 h. The first set of milk samples was analyzed for EC, pH [pen type conductometer–pHmeter (EZDO 7200)] and RI [handheld refractometer (RHB-32ATC, according to the brix scale] at 20 °C. Prior to analysis, the conductometer–pH meter was calibrated using standard conductivity solution (1413 μS/cm) and standard buffer solutions (pH = 4.0 and 7.0) according to the manufacturer’s instructions. An aliquot from this set of samples was transferred to 2 mL tubes and frozen at −18 °C for subsequent estimation of PL–PG system activity. The second set of samples was used to measure the SCC (FossomaticTM FC). Daily milk yield was calculated by adjusting the morning milking records according to ICAR-recommended methods (ICAR, 2018). Body condition score (BCS) was estimated for each ewe by the same veterinarian using a five-degree scale (1 = emaciated, 5 = obese).

### 2.3. Plasmin and Plasminogen Activity Estimation

Plasmin activity was determined without prior dissociation of the micelle-bound enzyme (e.g., by the use of ε-aminocaproic acid). Therefore, the measured activity reflects the readily available plasmin fraction measurable under the extraction conditions applied in the present assay rather than the total plasmin associated with casein micelles. Plasmin and plasminogen activities were determined using enzymatic assays. In detail, 900 μL of milk was mixed with 300 μL of 0.4 M trisodium citrate to promote casein micelle dispersion and facilitate recovery of the milk serum phase, and centrifuged at 27,000× *g* for 20 min. After the centrifugation, the cream layer was discarded and skimmed milk (500–800 μL) was collected for the following procedure. Plasmin activity was performed in a 100 μL reaction mixture containing 35 μL of 0.2 M Tris-HCl buffer pH 7.4, 35 μL of 0.86 mM D-Val-Leu-Lys p-nitroanilide dihydrochloride (V7127; Sigma Chemical Co., St. Louis, MO, USA), and 30 μL of milk sample. For plasminogen activity, the same reaction mixture was supplemented with 2 μL (12 Plough units) of urokinase (672112; Sigma Chemical Co., St. Louis, MO, USA). All reactions were performed in duplicate in 96-well flat-bottom polystyrene microtiter plates (Kisker Biotech GmbH & Co. KG, Steinfurt, Germany) and incubated at 37 °C. Distilled water was used as a negative control. Purified plasmin from human plasma (P1867; Sigma Chemical Co., St. Louis, MO, USA) was used as an activity reference standard and was included in each microplate to generate calibration curves and ensure inter-plate comparability of plasmin activity measurements. Absorbance was recorded at 405 nm at 20 min intervals for 160 min using a microplate photometer (Multiskan FC, Thermo Fisher Scientific Inc., Singapore). Plasmin and plasminogen activities were calculated from the linear portion of the absorbance-versus-time curves and expressed as assay-derived activity units per unit volume of milk. One unit of plasmin-derived activity was defined as the amount of enzyme producing a change in absorbance of 0.1 at 405 nm within 60 min under the assay conditions. Assay repeatability was assessed using duplicate measurements. Intra-assay coefficients of variation ranged between approximately 6 and 15% for both plasmin and plasminogen measurements. Inter-assay coefficients of variation were estimated using identical purified plasmin reference standards included in each microplate and were approximately 20% for plasmin and 25% for plasminogen. To account for potential plate-to-plate variation, activity values were normalized relative to the corresponding reference standards included in each plate.

### 2.4. Statistical Analyses

The statistical package for the social sciences (SPSS v26 software, IBM Corp., Armonk, NY, USA) was used for the statistical analyses and statistical significance was set at the 0.05 level. Our dataset included recordings for the total of 52 dairy ewes, each measured three times for the studied traits. SCC was log-transformed prior to analysis, the normality of variables was tested using the Kolmogorov–Smirnov test, and outliers were assessed using boxplots. Descriptive statistics (mean ± standard error) were calculated for the milk quantity and quality traits studied. One-way analysis of variance (ANOVA) was used to assess the differences in PL–PG system, electrical conductivity, refractive index, pH, and log-transformed SCC between Frizarta and Chios ewes for each time point, and non-parametric Spearman’s rank correlation coefficients were calculated for all milk traits, DMY, and BCS. Afterwards, a mixed linear regression model (separate for each breed) was formulated for the assessment of the effects of lactation stage, number of lactations, daily milk yield and SCC on the forementioned traits:

Y_jklm_ = μ + L_j_ + S_k_ + C_l_ + a_1_×DMY + E_m_ + δ_mk_ + e^jklm^ (model 1)

where Y_jklm_ represents the dependent variables (plasmin, plasminogen, plasmin+plasminogen activity, plasminogen:plasmin ratio, EC, RI, and pH of milk); μ = intercept; L_j_ = fixed effect of the lactation number (j = 2 levels; <3rd lactation and ≥3rd lactation); S_k_ = fixed effect of the sampling occasion (k = 3 levels; 3rd, 5th, and 6th month post-lambing); C_l_ = fixed effect of the SCC class (l = 4 levels; 1 = 0.0–0.5 (×10^6^ cells/mL), 2 = 0.5–1.0 (×10^6^ cells/mL), 3 = 1.0–3.0 (×10^6^ cells/mL), and 4 = >3.0 (×10^6^ cells/mL), a_1_ = fixed effect of the regression coefficient of daily milk yield-DMY); E_m_ = random variation in the m-th ewe; δ_ml_ = repeated variation in the m-th ewe in the k-th sampling occasion; e_jklm_ = residual error. Akaike’s information criterion (AIC) value was used to select the most appropriate covariance structure, and the first-order autoregressive was selected as the most appropriate one. Homoscedasticity was evaluated through residuals versus fitted values plots.

## 3. Results

**Figure 1:**
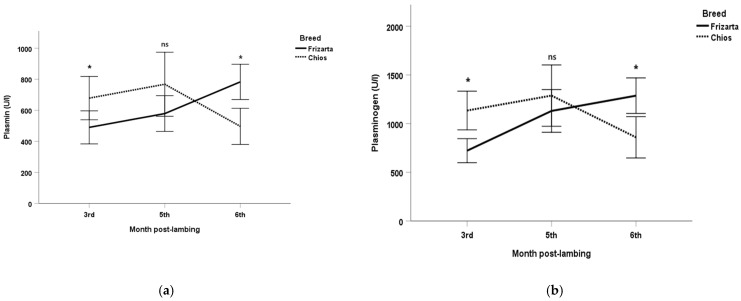

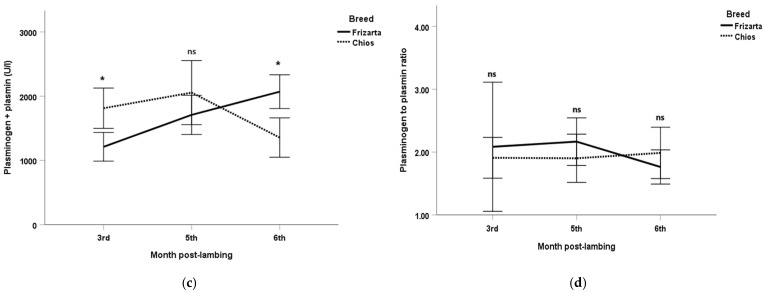
Mean values of (**a**) plasmin, (**b**) plasminogen, (**c**) plasmin plus plasminogen, (**d**) plasminogen-to-plasmin ratio for Frizarta and Chios ewes during the study (N = 52 ewes with 154 milk measurements). Error bars show 95% confidence intervals (CI) of means. *: statistically significant differences between breeds at each sampling occasion (*p* < 0.05); ns: not statistically significant differences between breeds at each sampling occasion.

**Figure 2:**
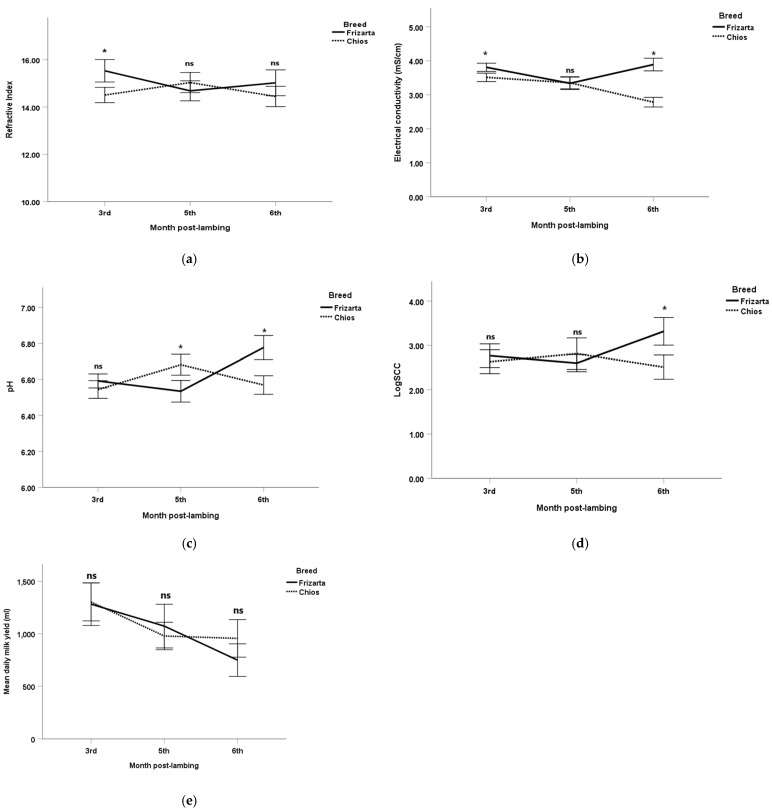
Mean values of (**a**) refractive index, (**b**) electrical conductivity, (**c**) pH, (**d**) logarithm of somatic cell counts, and (**e**) mean daily milk yield for Frizarta and Chios ewes during the study (N = 52 ewes with 154 milk measurements). Error bars show 95% confidence intervals (CI) of means. *: statistically significant differences between breeds at each sampling occasion (*p* < 0.05); ns: not statistically significant differences between breeds at each sampling occasion.

Mean plasmin activities ranged from 490 U/L (3rd month post-lambing) to 783 U/L (6th month post-lambing) for the Frizarta ewes and from 497 U/L (6th month post-lambing) to 768 U/L (5th month post-lambing) for the Chios ewes. The respective mean values for the plasminogen activities were from 722 U/L (3rd month post-lambing) to 1287 U/L (6th month post-lambing) and from 859 (6th month post-lambing) to 1288 U/L(5th month post-lambing) (Figure 1). Milk plasmin, plasminogen, and plasmin plus plasminogen activities were significantly higher in Chios ewes during the 3rd month post-lambing (*p* < 0.05, *p* < 0.001, and *p* < 0.01, respectively) and in Frizarta ewes during the 6th month post-lambing (*p* < 0.001, *p* < 0.01, and *p* < 0.001, respectively). Refractive index was significantly increased in the Frizarta ewes’ milk during the 3rd month post-lambing (*p* < 0.001) (Figure 2). Similarly, milk electrical conductivity was significantly higher in Frizarta ewes compared to Chios ewes during the 3rd month post-lambing (3.8 vs. 3.5, *p* < 0.001) and during the 6th month post-lambing (3.9 vs. 2.8, *p* < 0.001). pH was significantly increased in Chios compared to Frizarta ewes’ milk during the 5th month post-lambing (6.7 vs. 6.5, *p* < 0.001) and significantly decreased during the 6th month post-lambing (6.6 vs. 6.8, *p* < 0.001). The logarithm of SCC was significantly higher in Frizarta ewes’ milk during the 6th month post-lambing (*p* < 0.001). Also, Frizarta ewes had a higher BCS compared to Chios ewes both during the 3rd (2.9 vs. 2.5, *p* < 0.001) and the 5th (3.0 vs. 2.5, *p* < 0.001) month post-lambing, whereas during the 6th month post-lambing BCS had no statistically significant difference between the two breeds (2.9 vs. 2.8, *p* > 0.05). Plasminogen-to-plasmin ratio and daily milk yield were not significantly different between the two breeds at any time point. 

Plasmin was positively correlated with plasminogen (*p* < 0.001) and plasminogen plus plasmin (*p* < 0.01) but negatively associated with the plasminogen-to-plasmin ratio (*p* < 0.001) at every time point (Table 2). It was also positively correlated with EC (*p* < 0.001), pH (*p* < 0.001), LogSCC (*p* < 0.05) and BCS (*p* < 0.05), but only during the 6th month post-lambing. Moreover, plasminogen was positively correlated with plasminogen plus plasmin at all time points (*p* < 0.001) but not with the plasminogen-to-plasmin ratio, while it was positively correlated with EC during the 3rd and 6th month post-lambing (*p* < 0.05 and *p* < 0.001, respectively) and with pH (*p* < 0.01) only during the 6th month post-lambing. Also, plasminogen was negatively correlated with DMY (*p* < 0.05) during the 6th month post-lambing. Plasminogen-to-plasmin ratio was negatively correlated with EC (*p* < 0.05) during the 3rd month post-lambing and with pH (*p* < 0.05) during the 6th month post-lambing. Plasminogen plus plasmin were positively correlated with EC (*p* < 0.001) and pH (*p* < 0.01) and negatively associated with DMY (*p* < 0.05) during the 6th month post-lambing. In no case were any of the PL–PG system traits significantly correlated with RI.

In addition to the correlations described between the PL–PG system and EC, the latter was positively correlated with pH and LogSCC (*p* < 0.01) in all cases (except for the correlation with pH during the 5th month post-lambing which was not significant). Furthermore, pH was positively correlated (*p* < 0.001) with LogSCC during the 3rd and 6th month post-lambing. Refractive index was not significantly correlated with any of the other traits studied, with the exception of a negative correlation with EC (*p* < 0.01) observed during the 5th month post-lambing.

The overall effect of the month post-lambing was statistically significant in every case (*p* < 0.05), except for plasminogen in Frizarta ewes and plasminogen-to-plasmin ratio for both Frizarta and Chios ewes. Age was negatively associated only with plasmin in Frizarta ewes (*p* < 0.05), whereas DMY was negatively associated with plasminogen and plasminogen plus plasmin in Frizarta ewes (*p* < 0.05) and with the refractive index in both breeds. Somatic cell count was positively associated with electrical conductivity and pH in both breeds (*p* < 0.01) and negatively associated with the refractive index in Frizarta ewes (*p* < 0.05). The pairwise comparisons of all the explanatory variables for Frizarta and Chios ewes are presented in Table 3 and Table 4.

## 4. Discussion

The PL–PG system represents the major endogenous proteolytic pathway in milk and plays a pivotal role in casein degradation, milk technological properties, and mammary gland physiology. Activation of plasmin promotes casein degradation and is influenced by physiological and inflammatory processes in the mammary gland, which are also reflected in changes in milk physicochemical traits such as electrical conductivity and pH. In dairy ewes, its activation has been associated with both physiological changes during lactation and inflammatory processes affecting mammary gland integrity; however, published evidence remains limited and often inconsistent across breeds and production systems. By jointly evaluating PL–PG system activity, physicochemical milk traits, and SCC in intensively reared Chios and Frizarta ewes, the present study provides a results-driven assessment of the biological factors underlying endogenous proteolysis in ewe milk.

In the present study, lactation stage was consistently associated with plasmin system traits; however, the direction of this association differed markedly between breeds. In Chios ewes, plasmin and plasminogen plus plasmin activities were significantly higher during the 3rd and 5th month post-lambing compared with the reference 6th month, whereas in Frizarta ewes, lower values were observed at the same stages. This breed-dependent divergence indicates differences in the temporal regulation of endogenous proteolysis rather than a uniform lactation-stage effect. Such opposite lactation-stage patterns may reflect underlying physiological differences between breeds, including variations in mammary gland function, udder health dynamics, and metabolic regulation during lactation, in addition to farm-specific management and environmental effects. Importantly, the present results provide, to our knowledge, the first characterization of PL-PG system activity in the milk of Frizarta sheep, allowing breed-specific patterns of endogenous proteolysis to be evaluated within a high-yielding dairy sheep production context.

Similar variability has been reported in dairy sheep, where the lactation stage influences plasmin system activity but without a consistent pattern across breeds or flocks [7,8]. In Greek dairy sheep, Theodorou et al. [11] demonstrate that breed and lactation stage interactively affect PL–PG system activity, supporting the interpretation that chronological lactation stage alone does not fully explain proteolytic dynamics. Importantly, that study compared breeds with markedly different genetic potential for milk production, and part of the observed variation in PL–PG system parameters was interpreted in relation to differences in production capacity. When compared with previous studies, the absolute plasmin and plasminogen activity values observed in the present study are generally lower than those reported by Theodorou et al. [11] and Albenzio et al. [7,8]; this difference likely reflects methodological differences in the fraction of the plasmin system assessed rather than true biological discrepancies.

Evidence from dairy cows provides a useful comparative framework for interpreting these findings. Studies based on direct enzymatic measurements have consistently shown that, in cows, genetic background influences baseline PL-PG system activity, while the temporal pattern of plasmin activation across lactation is largely conserved among breeds and is characterized by a progressive increase toward late lactation and mammary involution [5,6,23]. Thus, in dairy cows, breed effects primarily modulate the magnitude of plasmin activity rather than the direction of its lactation-stage-related changes.

In contrast to both the heterogeneous-breed comparison performed by [11] and the conserved lactation-stage pattern reported in dairy cows, the present study focuses on two indigenous dairy sheep breeds that are both characterized by high milk-yield potential and are reared under comparable intensive, zero-grazing management conditions. This design minimizes confounding effects related to production level and allows breed-specific patterns of plasmin system regulation to be evaluated within a high-yielding production framework. The persistence of breed-dependent differences in PL–PG system activity observed here therefore suggests that intrinsic differences in mammary physiology and inflammatory responsiveness may exist even among highly productive breeds, independently of milk yield per se.

Somatic cell count emerges as a key determinant of milk traits and, particularly in Chios ewes, of plasmin system activity. In this breed, lower SCC classes (≤3.0 × 10^6^ cells/mL) are associated with significantly reduced plasmin and plasminogen+plasmin levels compared with the >3.0 × 10^6^ cells/mL reference, indicating a close link between plasmin system activation and mammary inflammatory status. This pattern supports the interpretation that, in Chios ewes, endogenous proteolysis is strongly driven by inflammation-related mechanisms.

These findings are in line with experimental evidence showing a strong association between SCC and plasminogen activator activity in sheep and goat milk, proposing proteolytic activity as a biological indicator of mammary gland health [10]. In dairy ewes, increased SCC has been repeatedly associated with enhanced plasmin activity and deterioration of cheese-making properties, underlining the technological relevance of inflammation-induced proteolysis [4,7,8]. Negative effects of elevated SCC on cheese yield and quality have also been documented in ewe milk independently of direct measurements of proteolytic activity [18]. In contrast, the weaker or inconsistent SCC-related effects observed in Frizarta ewes suggest breed-specific differences in inflammatory responsiveness or in the regulation of protease activation—a phenomenon also suggested by comparative studies among sheep breeds [11].

Beyond the plasmin system, several indigenous proteolytic enzymes contribute to milk protein turnover in ovine and caprine milk and interact with physiological and inflammatory signals during lactation [21]. The mechanisms underlying plasmin system activation appear to involve both cellular and structural components of the mammary gland. Although somatic cells are often implicated in protease-related changes, their direct contribution to plasmin activity may be limited. Caroprese et al. [24] report that macrophages contribute minimally to overall plasmin activity in ewe bulk milk, despite increased plasminogen activator activity during lactation. This suggests that alterations in epithelial barrier integrity and enhanced transfer of blood-derived enzymes play a dominant role. Mechanistic support for this interpretation is provided by goat studies demonstrating that somatic cell-associated processes contribute to plasminogen activation and subsequent caseinolysis within the mammary gland [25]. Taken together, these findings indicate that SCC-related plasmin system activation reflects a combination of inflammatory cell presence and permeability-related mechanisms rather than direct secretion of active proteases alone, which may explain the breed-dependent SCC effects observed in the present study.

Within this mechanistic context, an additional noteworthy finding of the present study is the relative stability of the plasminogen-to-plasmin (PG:PL) ratio across lactation stages, breeds, and somatic cell count classes. Despite marked variation in absolute plasmin and plasminogen activity levels, their ratio remained largely unchanged, indicating that activation of the PL-PG system occurred in a coordinated and proportionate manner. This pattern suggests that the observed changes in endogenous proteolytic activity primarily reflect quantitative modulation of the system rather than disproportionate or dysregulated conversion of plasminogen to plasmin. Similar observations have been reported in dairy sheep, where parallel changes in plasmin and plasminogen activities result in limited variation in their ratio under physiological and moderate inflammatory conditions [11]. Collectively, these findings support the concept that the PL-PG system in ewe milk is tightly regulated, with maintenance of a stable PG:PL balance potentially acting as a safeguard against excessive proteolysis that could compromise mammary tissue integrity and milk technological properties.

Within the context of coordinated plasmin system regulation, the association between BCS and plasmin activity appears to be stage-specific. Although BCS is not consistently associated with plasmin system traits across lactation, a positive correlation between BCS and PL activity was detected during the 6th month post-lambing. This finding indicates that metabolic status may modulate inter-individual variation in plasmin activity during late lactation without determining overall system activation.

Differences in BCS trajectories between breeds further contribute to the interpretation of breed-specific plasmin system patterns. Frizarta ewes exhibit relatively stable BCS values across the examined stages of lactation, whereas Chios ewes show a more pronounced increase in BCS toward the 6th month post-lambing. These contrasting patterns suggest variation in metabolic adaptation during late lactation, which may influence mammary gland physiology and contribute to the divergent plasmin system trajectories observed between breeds. Collectively, the BCS-related findings indicate that metabolic status modulates plasmin activity in a context-dependent manner, without overriding the primary effects of lactation stage and mammary health status.

Electrical conductivity (EC) and pH show clear associations with lactation stage and SCC in both breeds, reinforcing their value as indirect indicators of mammary gland integrity. In the present data, EC increased significantly during mid-lactation in Chios ewes and was consistently lower at low SCC classes, reflecting changes in milk ionic composition associated with inflammatory status. Increased EC is a well-established consequence of mastitis-related disruption of tight junctions and altered ion transport, leading to elevated sodium and chloride concentrations in milk [12]. Milk pH follows similar SCC-related patterns, with lower pH values observed at lower SCC classes. Although pH alone has limited diagnostic value for subclinical mastitis, increased pH can influence enzyme activity, casein micelle stability, and calcium balance [14]. These changes can impair rennet coagulation and curd formation, ultimately leading to reduced cheese-making capacity [26,27]. Comparable effects of lactation stage and SCC on milk physicochemical properties have been reported in dairy ewes [16,17], supporting the interpretation that EC and pH act as sensitive, complementary indicators of mammary health-related milk alterations.

The refractive index (RI) was included in the present study as a complementary indicator of milk compositional status in relation to proteolytic activity. Unlike conventional milk composition parameters such as fat, protein, or total solids, which require laboratory-based analyses, RI can be measured rapidly with portable refractometers, therefore representing a practical tool for on-farm milk quality assessment in combination with EC and pH measurement. According to our results, RI is affected by lactation stage and SCC but shows limited association with plasmin system traits, particularly in Frizarta ewes. This finding suggests that RI primarily reflects compositional changes in milk solids rather than endogenous proteolytic activity. Previous studies have shown that RI can predict milk fat, protein, lactose, and total solids content in ovine milk, especially when combined with EC and pH [15]. The weak association between RI and PL–PG traits observed here indicates that compositional modulation and enzymatic proteolysis are partially uncoupled processes.

Results from goat milk studies provide additional context for interpreting the present findings. Fantuz et al. [28] demonstrated that elevated plasmin and plasminogen activator activity in late-lactating goats resulted in increased casein degradation and impaired coagulation properties, confirming that excessive endogenous proteolysis compromises milk technological quality. Moreover, species-specific differences in plasmin system activation during subclinical mastitis have been reported, with sheep appearing more susceptible to proteolysis-related milk quality deterioration than goats [29]. These observations reinforce the importance of species- and breed-specific interpretation of plasmin system activity.

Daily milk yield did not exert a biologically meaningful effect on plasmin system traits or physicochemical parameters once lactation stage and SCC were accounted for, except for plasminogen and plasminogen plus plasmin in Frizarta ewes. Specifically, increasing DMY was associated with decreasing activities of plasminogen and plasminogen plus plasmin in Frizarta ewes, indicating that, in this breed, endogenous milk proteolysis may also be influenced by production level in addition to physiological stage and mammary health. In a similar study, Theodorou et al. [11] reported no relationship between plasmin activity and milk yield despite marked differences in production capacity among Greek dairy sheep breeds (mixed indigenous breed and Chios breed), which is consistent with the absence of the DMY effect observed in Chios ewes in our study. Notably, Frizarta is a composite breed derived mainly from foreign genetic material, which may partly explain this differentiated response compared with the indigenous Chios breed.

Parity effects were limited, indicating that within the parity range examined, mammary health status and lactation-related physiological changes were more influential determinants of milk proteolysis and physicochemical variation than age alone. Importantly, the breed-specific patterns observed throughout this study highlight that interpretation of plasmin system measurements and the establishment of reference thresholds for milk quality assessment should be based on breed-adapted baselines rather than generalized criteria.

However, a limitation of the present study is that each breed was reared on a single farm (Frizarta breed in Farm A and Chios breed in Farm B). Although the farms were located in the same region and applied similar intensive management systems and production methods, the effects of breed and farm cannot be completely separated. Therefore, the differences observed between breeds may not be attributed exclusively to genetic differences but also to farm-specific environmental or management effects. More farms and animals from these dairy sheep breeds should be included in future studies to better disentangle breed and farm effects on the PL-PG system. Furthermore, future studies integrating milk composition and detailed casein fraction profiles with the PL–PG system would be valuable for further elucidating the biochemical mechanisms underlying the associations observed in the present study.

## 5. Conclusions

The present study demonstrates that activation of the PL-PG system in ovine milk is primarily governed by lactation stage and mammary health status, as reflected by SCC, rather than by daily milk yield. Breed-specific patterns were identified, with Chios and Frizarta ewes exhibiting contrasting lactation-stage trajectories and differing sensitivity of plasmin system activity to SCC, highlighting the importance of genetic background in modulating endogenous proteolysis. Body condition score emerged as a secondary, stage-dependent modifier of plasmin activity during late lactation, contributing to inter-individual variation without overriding the dominant effects of lactation stage and mammary health. Physicochemical traits, particularly EC and pH, were closely associated with SCC and provided complementary information on mammary gland integrity. In this context, routinely measured indicators such as SCC, EC, and pH, together with PL-PG system activity, may provide useful complementary tools for monitoring milk quality and assessing milk-processing suitability in intensive dairy sheep systems. Overall, these findings indicate that endogenous proteolysis in ovine milk arises from complex interactions between physiological stage, inflammatory status, and breed-specific mammary characteristics, with direct implications for milk quality assessment and cheese-making performance in intensive dairy sheep systems.

## Figures and Tables

**Table 1:** Structural and management characteristics of Farms A and B as declared by the farmers and observed during the study.

Livestock	Farm A	Farm B
Breed of sheep	Frizarta	Chios
Adult ewes, n	340	250
Rams, n	20	12
Yearlings, n	100	50
Ewes to bucks ratio	16:1	21:1
Rams’ replacement rate, %	33	25
Yearlings to ewes ratio, %	34	20
Ewes’ body weight, kg	70	60
Rams’ body weight, kg	90	85
Age at first mating, months	9	9
**Production**		
Milk production/ewe/lactation (210 days), kg	340	330
Average milk fat content (%)	7.3	6.8
Average milk protein content (%)	6.2	5.7
Average somatic cell count (cells/mL)	0.5 × 10^6^ to 1.0 × 10^6^	0.5 × 10^6^ to 1.0 × 10^6^
Average total bacterial count (bacteria/mL)	100 × 10^3^ to 150 × 10^3^	50 × 10^3^ to 100 × 10^3^
**Management**		
Duration of milking period (d)	225	230
Duration of suckling period (d)	50	50
Duration of dry period (d)	90	85
Type of milking	Machine milking	Machine milking
Number of milkings per day	2	2
Post-dipping of teats	No	No
Surface per ewe (m^2^)	1.6	1.5
**Reproduction**		
Lambing season	October	November
Prolificacy (lambs/ewe at birth)	1.5	1.8
Lambing interval (months)	12	12
Culling rate due to infertility (%)	5	7
Average litter size at weaning	1.3	1.4
**Feeding and nutrition**		
Feedings per day	3	2
Grazing	No	No
Concentrates in milking ewes (kg/day)	0.9–1.3	0.9–1.3
Roughages (alfalfa hay) in milking ewes (kg/day)	1.0–1.3	1.0–1.3
Roughages (barley straw) in milking ewes	Ad lib	Ad lib
**Health and welfare**		
Sick animals during visits (%)	<5	<5
Clinical acidosis incidence (%)	≤2	≤2
Pregnancy toxemia incidence (%)	≤2	≤2
Abortion incidence (%)	≤3	≤5
Retained placenta incidence (%)	≤2	≤2
Metritis incidence (%)	≤3	≤2
Clinical mastitis incidence (%)	≤10	≤8
Subclinical mastitis incidence (%)	≤25	≤20
Lameness incidence (%)	≤5	≤3
Other diseases sporadically observed	Contagious ecthyma (orf), copper toxicity	Enterotoxaemia, Maedi-Visna

**Table 2:** Correlation table of the plasmin–plasminogen system traits, daily milk yield, refractive index, electrical conductivity, pH, the logarithm of somatic cell counts, and the body condition score of the studied ewes.

	Trait	PL	PG	PG:PL	PG+PL	DMY	RI	EC	pH	LogSCC	BCS
3rd month post-lambing	PL	1									
	PG	0.769 ***	1								
	PG:PL	−0.602 ***	−0.009	1							
	PG+PL	0.916 **	0.951 ***	−0.279 *	1						
	DMY	−0.181	−0.210	0.097	−0.219	1					
	RI	−0.035	−0.095	−0.040	−0.050	−0.124	1				
	EC	−0.034	−0.350 *	−0.330 *	−0.244	0.191	−0.110	1			
	pH	−0.201	−0.216	0.124	−0.219	0.128	−0.187	0.393 **	1		
	LogSCC	0.015	−0.068	−0.104	−0.028	−0.013	−0.030	0.387 **	0.527 ***	1	
	BCS	−0.088	−0.211	−0.079	−0.141	0.032	0.261	0.224	0.160	0.137	1
5th month post-lambing	PL	1									
	PG	0.738 ***	1								
	PG:PL	−0.590 ***	−0.056	1							
	PG+PL	0.887 ***	0.952 ***	−0.192	1						
	DMY	−0.139	−0.231	−0.010	−0.224	1					
	RI	0.117	0.166	0.051	0.165	0.081	1				
	EC	0.006	0.085	0.023	0.041	−0.244	−0.398 **	1			
	pH	0.152	0.120	−0.154	0.137	−0.364 **	−0.018	−0.032	1		
	LogSCC	0.197	0.036	−0.218	0.050	−0.122	0.028	0.366 *	0.094	1	
	BCS	0.011	0.006	0.042	0.015	−0.066	−0.180	−0.003	−0.278	0.012	1
6th month post-lambing	PL	1									
	PG	0.722 ***	1								
	PG:PL	−0.459 ***	0.056	1							
	PG+PL	0.879 ***	0.957 ***	−0.030	1						
	DMY	−0.271	−0.323 *	0.064	−0.311 *	1					
	RI	0.139	0.093	−0.006	0.144	−0.056	1				
	EC	0.469 ***	0.446 ***	−0.106	0.481 ***	−0.399 **	0.091	1			
	pH	0.485 ***	0.356 **	−0.287 *	0.429 **	−0.402 **	−0.017	0.707 ***	1		
	LogSCC	0.288 *	0.210	−0.153	0.242	−0.408 *	0.173	0.624 ***	0.664 ***	1	
	BCS	0.275 *	−0.009	−0.269	0.096	−0.105	−0.024	0.268	−0.346 *	0.135	1

PL: plasmin; PG: plasminogen: PG:PL: plasminogen-to-plasmin ratio; DMY: daily milk yield; RI: refractive index; EC: electrical conductivity: LogSCC: logarithm of somatic cell counts; BCS: body condition score: *** *p* ≤ 0.001; ** *p* ≤ 0.01; * *p* ≤ 0.05.

**Table 3:** Effects of lactation stage, age, daily milk yield, and somatic cell counts on plasmin and plasminogen activities system in Frizarta and Chios ewes.

Milk Trait		Frizarta Ewes		Chios Ewes	
	Parameter	Estimate ± SE	*p*	Estimate ± SE	*p*
Plasmin (U/L)	3rd month post-lambing	−221.9 ± 81.06	0.008	209.0 ± 103.69	0.048
	5th month post-lambing	−198.0 ± 82.21	0.020	268.5 ± 114.50	0.024
	<3rd lactation	179.3 ± 77.12	0.026	136.2 ± 88.01	0.130
	0.0–0.5 (×10^6^) cells/mL	31.3 ± 84.56	0.713	−265.0 ± 121.79	0.034
	0.5–1.0 (×10^6^) cells/mL	164.9 ± 114.13	0.153	−351.6 ± 181.68	0.058
	1.0–3.0 (×10^6^) cells/mL	85.1 ± 97.79	0.387	−376.9 ± 153.45	0.017
	Daily milk yield (L)	−115.6 ± 65.52	0.086	1.1 ± 103.32	0.992
Plasminogen (U/L)	3rd month post-lambing	−434.9 ± 129.01	0.001	305.8 ± 155.43	0.054
	5th month post-lambing	−177.9 ± 114.31	0.126	347.8 ± 162.24	0.038
	<3rd lactation	157.3 ± 143.13	0.280	166.8 ± 142.23	0.250
	0.0–0.5 (×10^6^) cells/mL	212.9 ± 139.86	0.133	−206.0 ± 188.55	0.279
	0.5–1.0 (×10^6^) cells/mL	461.8 ± 173.18	0.010	−336.3 ± 273.46	0.224
	1.0–3.0 (×10^6^) cells/mL	203.1 ± 156.27	0.198	−393.5 ± 235.38	0.100
	Daily milk yield (L)	−270.8 ± 117.39	0.026	18.4 ± 158.17	0.908
Plasminogen + Plasmin (U/L)	3rd month post-lambing	−652.7 ± 190.22	0.001	507.5 ± 241.61	0.040
	5th month post-lambing	−374.8 ± 177.22	0.039	617.8 ± 255.70	0.020
	<3rd lactation	331.3 ± 196.93	0.102	307.9 ± 216.47	0.164
	0.0–0.5 (×10^6^) cells/mL	252.4 ± 202.71	0.218	−471.2 ± 290.80	0.111
	0.5–1.0 (×10^6^) cells/mL	634.8 ± 261.07	0.018	−690.0 ± 424.53	0.109
	1.0–3.0 (×10^6^) cells/mL	296.8 ± 230.23	0.202	−751.0 ± 363.59	0.044
	Daily milk yield (L)	−396.4 ± 164.94	0.021	32.1 ± 244.25	0.896
Plasminogen:plasmin ratio	3rd month post-lambing	0.23 ± 0.518	0.658	−0.07 ± 0.277	0.798
	5th month post-lambing	0.22 ± 0.462	0.634	−0.13 ± 0.308	0.671
	<3rd lactation	−0.54 ± 0.568	0.348	−0.16 ± 0.234	0.510
	0.0–0.5 (×10^6^) cells/mL	0.77 ± 0.560	0.177	0.52 ± 0.325	0.114
	0.5–1.0 (×10^6^) cells/mL	0.51 ± 0.698	0.470	0.51 ± 0.485	0.302
	1.0–3.0 (×10^6^) cells/mL	0.14 ± 0.627	0.819	0.61 ± 0.409	0.142
	Daily milk yield (L)	−0.18± 0.468	0.707	−0.12 ± 0.276	0.678

Reference categories: (i) for the stage of lactation (months post-lambing): 6th month post-lambing, (ii) for the lactation number: ≥3rd lactation, and (iii) for the SCC class: >3.0 (×10^6^) cells/mL. Intercept values for plasmin, plasminogen, plasminogen+plasmin, plasminogen:plasmin ratio for Frizarta and Chios ewes were: 787.0 U/L, 1294.1 U/L, 2084.1 U/L, 1.67, and 680.7 U/L, 974.9 U/L, 1640.3 U/L, 1.71, respectively. SE: standard error; *p*: *p*-value.

**Table 4:** Effects of lactation stage, age, daily milk yield, and somatic cell counts on electrical conductivity, refractive index and pH of milk in Frizarta and Chios ewes.

Milk Trait		Frizarta Ewes		Chios Ewes	
	Parameter	Estimate ± SE	*p*	Estimate ± SE	*p*
Electrical conductivity (mS/cm)	3rd month post-lambing	0.02 ± 0.113	0.827	0.68 ± 0.070	0.000
	5th month post-lambing	−0.40 ± 0.094	0.000	0.51 ± 0.061	0.000
	<3rd lactation	−0.00 ± 0.138	0.995	0.10 ± 0.111	0.356
	0.0–0.5 (×10^6^) cells/mL	−0.48 ± 0.124	0.000	−0.35 ± 0.093	0.000
	0.5–1.0 (×10^6^) cells/mL	−0.45 ± 0.145	0.003	−0.17 ± 0.126	0.189
	1.0–3.0 (×10^6^) cells/mL	−0.24 ± 0.137	0.082	−0.01 ± 0.118	0.937
	Daily milk yield (L)	−0.02 ± 0.108	0.844	0.04 ± 0.084	0.635
Refractive index	3rd month post-lambing	0.64 ± 0.331	0.059	0.27 ± 0.246	0.279
	5th month post-lambing	−0.44 ± 0.265	0.106	0.72 ± 0.227	0.003
	<3rd lactation	−0.92 ± 0.460	0.057	−0.44 ± 0.300	0.152
	0.0–0.5 (×10^6^) cells/mL	1.14 ± 0.375	0.003	−0.82 ± 0.317	0.012
	0.5–1.0 (×10^6^) cells/mL	0.77 ± 0.417	0.070	−0.33 ± 0.439	0.455
	1.0–3.0 (×10^6^) cells/mL	0.52 ± 0.400	0.201	−0.70 ± 0.398	0.084
	Daily milk yield (L)	−0.71 ± 0.3337	0.039	−0.56 ± 0.275	0.046
pH	3rd month post-lambing	−0.14 ± 0.034	0.000	−0.02 ± 0.033	0.534
	5th month post-lambing	−0.19 ± 0.027	0.000	0.14 ± 0.032	0.000
	<3rd lactation	−0.02 ± 0.045	0.678	0.00 ± 0.034	0.974
	0.0–0.5 (×10^6^) cells/mL	−0.17 ± 0.038	0.000	−0.15 ± 0.041	0.000
	0.5–1.0 (×10^6^) cells/mL	−0.13 ± 0.042	0.003	−0.13 ± 0.058	0.030
	1.0–3.0 (×10^6^) cells/mL	−0.09 ± 0.040	0.037	−0.11 ± 0.051	0.035
	Daily milk yield (L)	−0.02 ± 0.035	0.603	−0.01 ± 0.035	0.673

Reference categories: (i) for the stage of lactation (months post-lambing): 6th month post-lambing, (ii) for the lactation number: ≥3rd lactation, and (iii) SCC class: >3.0 (×10^6^) cells/mL. Intercept values for electrical conductivity, refractive index, and pH for Frizarta and Chios ewes are 4.16, 15.18, 6.88 and, 2.95, 15.80, and 6.71, respectively. SE: standard error; *p*: *p*-value.

## Data Availability

Data is available at https://doi.org/10.6084/m9.figshare.31325338.

## Abbreviations

The following abbreviations are used in this manuscript:

PL	Plasmin
PG	Plasminogen
PL+PG	Plasmin plus plasminogen
PG:PL	Plasminogen-to-plasmin ratio
SCC	Somatic cell count
BCS	Body condition score
EC	Electrical conductivity
RI	Refractive index
